# Acquired Distichiasis in Dermatomyositis: A Possible Delayed Sequela of Chronic Periocular Inflammation

**DOI:** 10.7759/cureus.115306

**Published:** 2026-08-27

**Authors:** Kawtar El Fid, Meryem Soughi, Hanae Lammini, FatimaZahra Mernissi

**Affiliations:** 1 Department of Dermatology, Hassan II University Hospital, Fez, MAR

**Keywords:** acquired distichiasis, dermatomyositis, eyelid dermoscopy, meibomian glands, periocular inflammation

## Abstract

Acquired distichiasis is an uncommon eyelid disorder characterized by an accessory row of eyelashes arising from the meibomian gland orifices, most often in the setting of chronic inflammatory or cicatricial disease. We report a case of a 65-year-old woman with longstanding dermatomyositis, recurrent periocular inflammation, and episodes of blepharitis who developed bilateral upper-eyelid distichiasis. Review of clinical photographs from 2020 and from a severe periocular flare in 2023 showed no clearly visible accessory row. At follow-up in January 2026, clinical and dermoscopic examination demonstrated bilateral accessory rows of long, pigmented eyelashes, findings consistent with acquired distichiasis. The patient had no ocular irritation or visual symptoms, and ophthalmologic evaluation found neither trichiasis nor corneal involvement. There was no history of Stevens-Johnson syndrome, ocular cicatricial pemphigoid, trachoma, chemical injury, or eyelid surgery. However, recurrent blepharitis and previous exposure to docetaxel in 2018 were important alternative or contributing factors. Chronic periocular inflammation associated with dermatomyositis may have promoted eyelid margin remodeling and metaplastic changes in the meibomian glands, but a causal relationship cannot be established from a single case. Because the patient was asymptomatic and had no corneal complications, observation with regular ophthalmologic monitoring was selected. This case highlights the need to examine the eyelid margins in patients with chronic periocular inflammation and to consider both inflammatory and treatment-related contributors to acquired distichiasis.

## Introduction

Acquired distichiasis (di=two; stichos=row) is an uncommon eyelid disorder characterized by an accessory row of eyelashes arising from the meibomian gland orifices [1]. It differs from congenital distichiasis, which is present from birth or develops early in life and may be associated with inherited syndromes, and from trichiasis, in which normally positioned eyelashes are misdirected toward the ocular surface. The acquired form usually develops after chronic inflammation or scarring of the eyelid margin, conjunctiva, or tarsal plate. Metaplasia and dedifferentiation of meibomian glands into hair-producing pilosebaceous units have been proposed as the underlying mechanism [2,3].

Reported associations include ocular cicatricial pemphigoid, Stevens-Johnson syndrome, trachoma, chronic rosacea, long-standing blepharitis, trauma, and previous eyelid surgery [3]. Rare treatment-related cases have also been described after systemic anticancer therapy [4]. Repeated eyelid margin inflammation may promote tissue remodeling and metaplastic change in the meibomian glands. We report bilateral acquired distichiasis in a patient with long-standing dermatomyositis and recurrent periocular inflammation. This possible association may be clinically relevant because accessory eyelashes can injure the ocular surface. Nevertheless, recurrent blepharitis and previous docetaxel exposure remain important competing or contributing explanations.

## Case presentation

A 65-year-old woman had been followed in our dermatology department since 2013 for dermatomyositis confirmed clinically and histologically. Her initial disease course included characteristic cutaneous findings together with proximal myalgias, fatigability, and weakness. During more recent follow-up, no objective muscle weakness was identified, and serial creatine kinase and aldolase levels remained within normal limits.

Her medical history included type 2 diabetes mellitus, gastric diffuse large B-cell lymphoma treated with six cycles of rituximab, cyclophosphamide, doxorubicin, vincristine, and prednisone (R-CHOP), with the last cycle administered in January 2016 and subsequent remission, and triple-negative invasive ductal breast carcinoma. The breast carcinoma was treated by mastectomy in 2018 followed by three cycles of fluorouracil, epirubicin, and cyclophosphamide (FEC100), three cycles of docetaxel, and radiotherapy; chemotherapy was completed approximately in October 2018.

The dermatologic course was marked by photosensitive flares with violaceous erythema of the eyelids and malar areas, poikilodermatous erythema of the neckline, Gottron papules, and erythematous, scaly, alopecic plaques of the scalp. Recurrent periocular erythema and episodes of blepharitis were documented. Treatments over the disease course included systemic corticosteroids, cyclophosphamide boluses, methotrexate, topical anti-inflammatory agents, and strict photoprotection.

**Figure 1:**
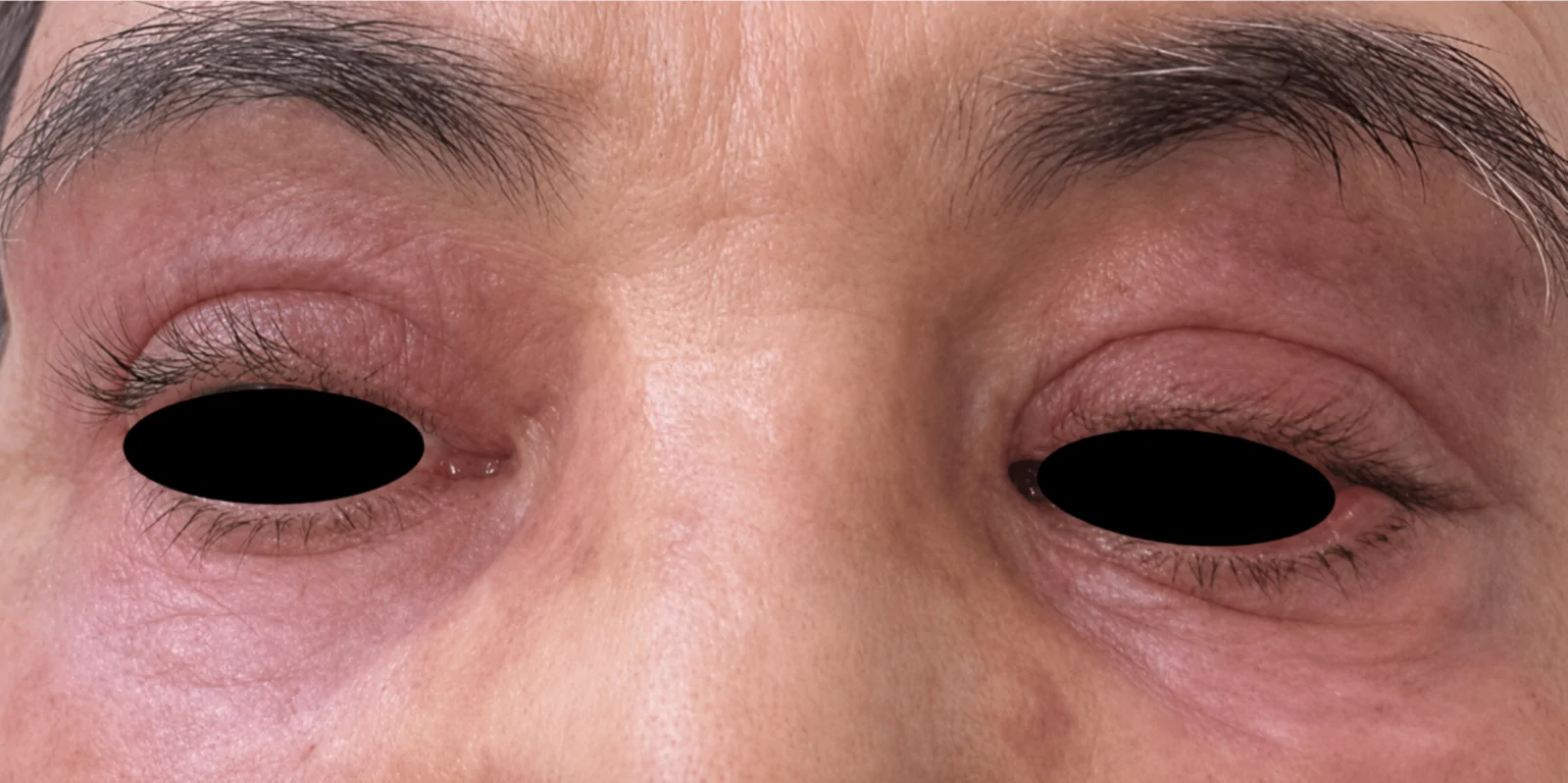
Bilateral eyelid examination in 2020 showing no evidence of distichiasis prior to dermatological disease flare.

**Figure 2:**
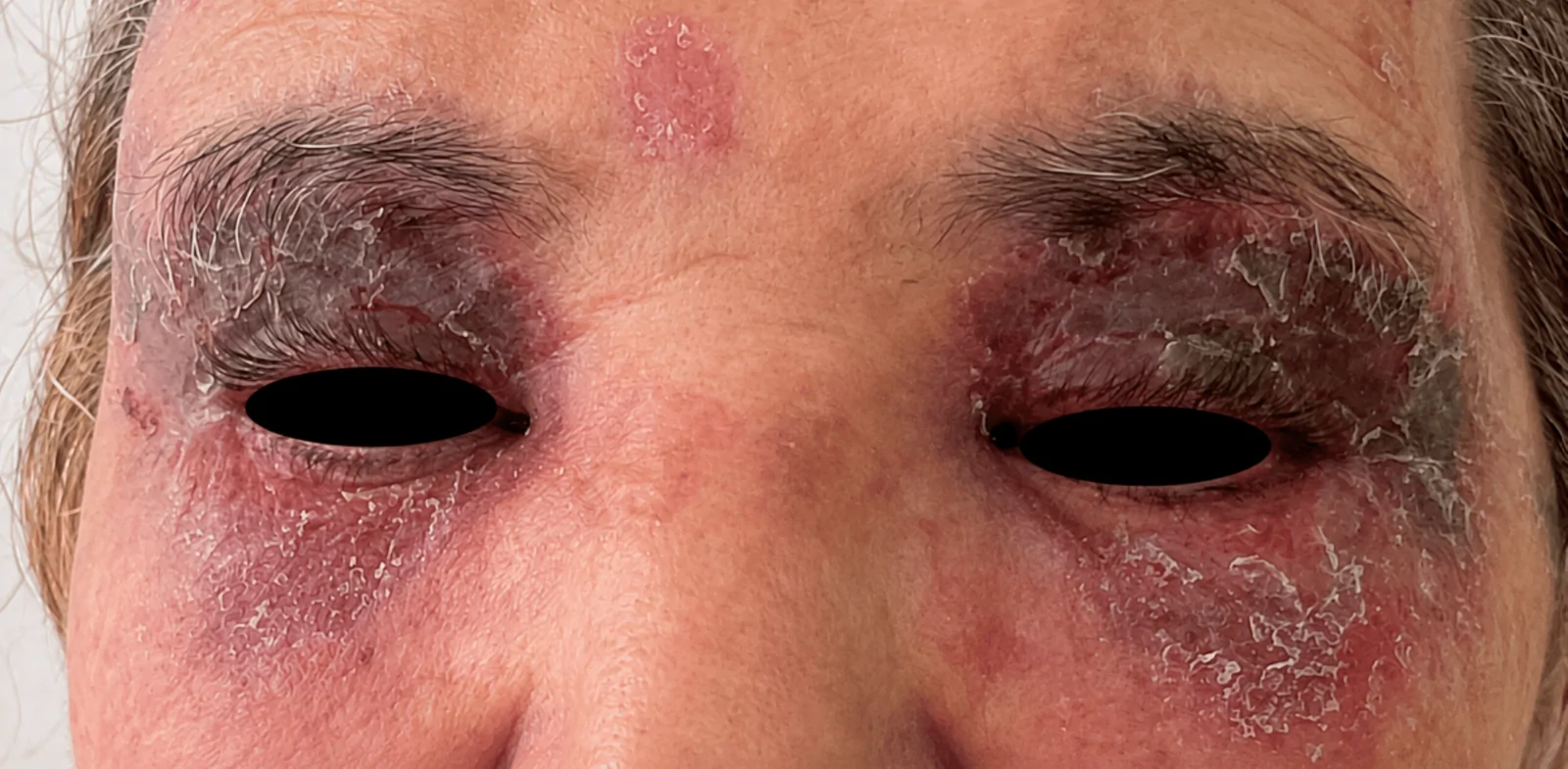
Severe periocular dermatomyositis flare in 2023. Bilateral eyelid examination in 2023 during a severe cutaneous flare of dermatomyositis showed erythematous-violaceous scaly plaques in the periorbital region, consistent with active cutaneous involvement, without evidence of distichiasis.

Review of a clinical photograph from 2020 showed no clearly visible accessory row of eyelashes (Figure 1). During a severe cutaneous flare in 2023, marked bilateral violaceous and scaly periocular inflammation was present, but an accessory row was not clearly visible on the available photograph (Figure 2). These retrospective images did not permit detailed visualization of every meibomian gland orifice.

**Figure 3:**
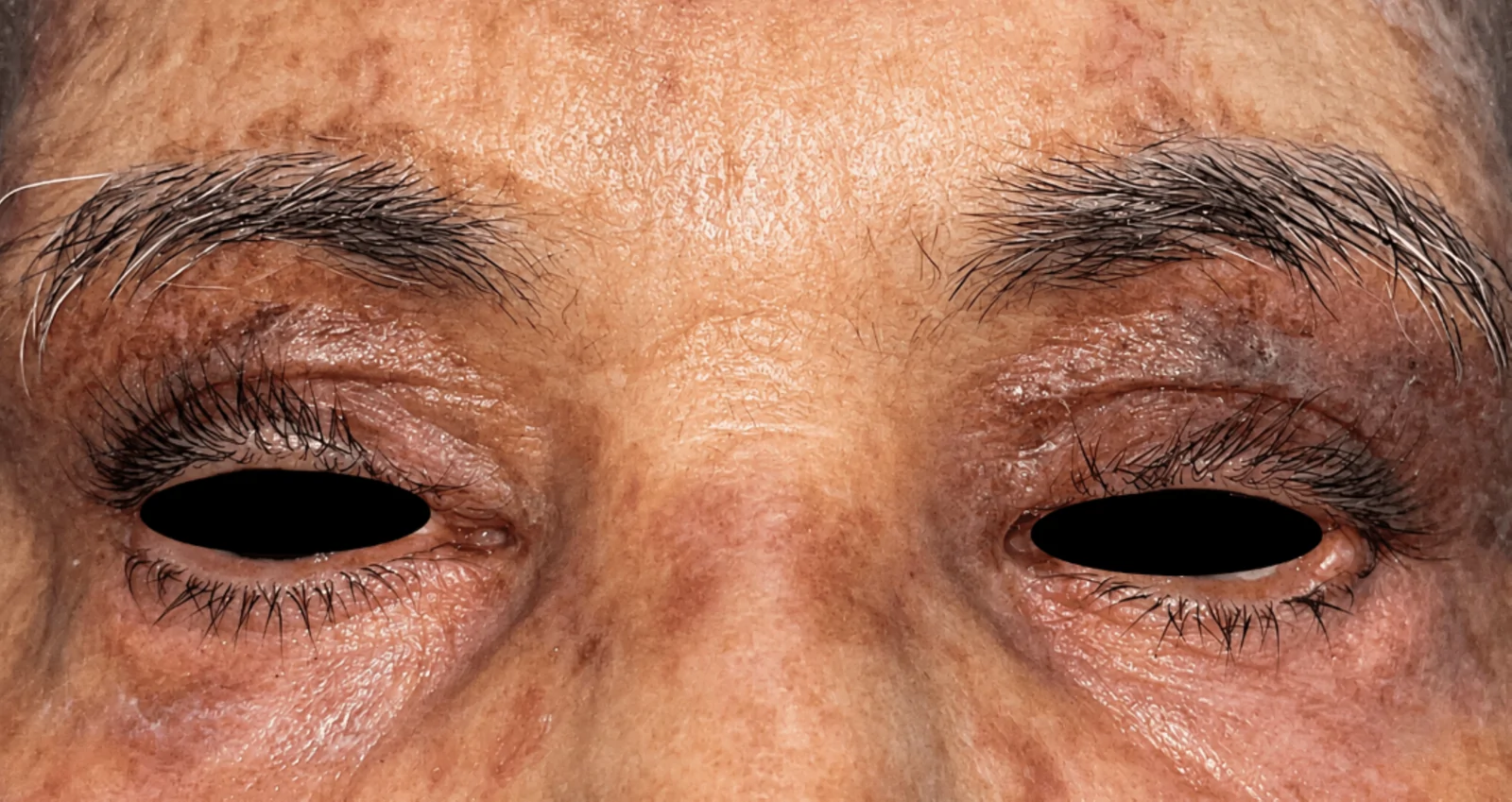
Upper eyelid examination in 2026 showing bilateral acquired distichiasis, associated with residual erythema and early post-inflammatory hyperpigmentation, reflecting partial dermatological remission.

**Figure 4:**
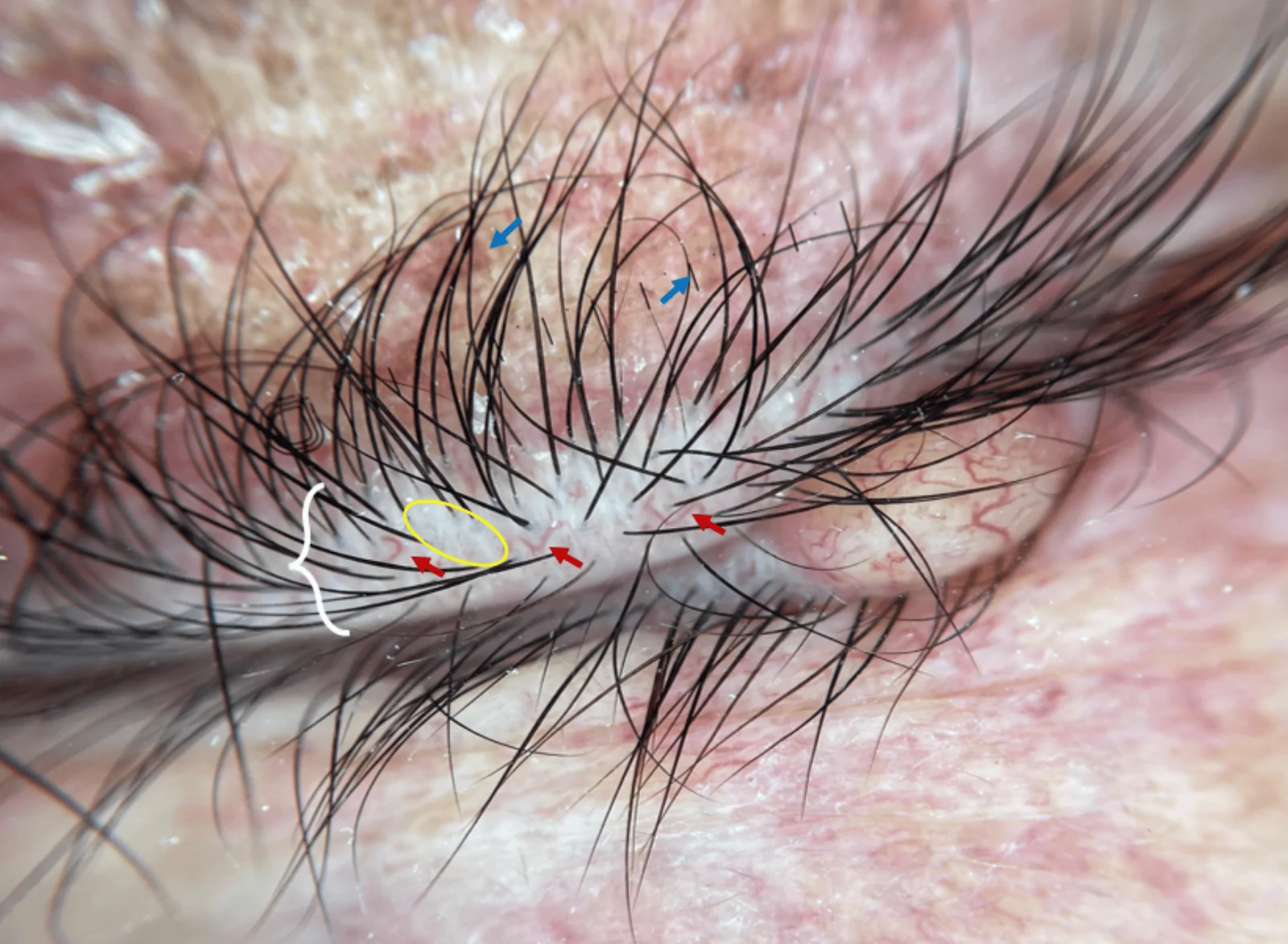
Dermoscopy of the upper eyelid showing multiple rows of long, pigmented eyelashes emerging from the eyelid margin, consistent with acquired distichiasis (white bracket). The underlying skin shows structureless white areas (yellow circle), telangiectasias (red arrow), and an erythematous background with a focal pseudopigment network (blue arrow).

At follow-up in January 2026, bilateral accessory rows of long, pigmented eyelashes were visible along the upper eyelid margins, consistent with acquired distichiasis (Figure 3). Chronic residual periocular erythema and hyperpigmentation were also present. The patient remained asymptomatic, without foreign-body sensation, ocular irritation, or visual complaints. Ophthalmologic evaluation showed no corneal involvement and no associated trichiasis. Dermoscopy demonstrated multiple rows of long, pigmented eyelashes along the eyelid margin. Associated findings included structureless white areas, telangiectasias, an erythematous background, and a focal pseudopigment network (Figure 4).

Based on the clinical and dermoscopic findings, a diagnosis of bilateral acquired distichiasis was made. There was no history of Stevens-Johnson syndrome, ocular cicatricial pemphigoid, trachoma, chemical injury, or previous eyelid surgery. Because the patient had no symptoms or ocular-surface complications and preferred to avoid intervention, no lash-directed treatment was undertaken. Regular dermatologic and ophthalmologic monitoring was planned.

## Discussion

Acquired distichiasis is primarily associated with chronic inflammatory or cicatricial disorders of the eyelid [3,5,6]. In our patient, recurrent periocular inflammation and episodes of blepharitis accompanied the course of dermatomyositis over several years. Persistent inflammation involving the eyelid margin, conjunctiva, and tarsal plate may induce progressive tissue remodeling and metaplastic transformation of the meibomian glands into hair-producing pilosebaceous units [2,3,5]. Dermatomyositis is characterized by sustained immune activation, type I interferon signaling, and cytokine-mediated tissue injury [7]. A recent review of ocular and eyelid involvement in dermatomyositis further emphasizes that the disease may affect periocular and ocular structures [8]. Repeated periocular flares may therefore have created a local inflammatory environment conducive to the development of accessory eyelashes, supporting the hypothesis of a possible association between dermatomyositis and acquired distichiasis.

Previous systemic anticancer therapy may also have contributed to this process. Distichiasis associated with entropion has been reported during combined treatment with pertuzumab, trastuzumab, and docetaxel [4]. More recently, a prospective study of breast cancer patients receiving docetaxel demonstrated narrowing and/or atrophy of the meibomian glands on meibography [9]. Chemotherapy-related tearing has also been associated with meibomian gland dysfunction, in addition to lacrimal drainage obstruction [10]. These findings support the biological plausibility of treatment-related alterations of the eyelid margin and meibomian glands. Our patient received docetaxel-containing chemotherapy in 2018, several years before the recognition of distichiasis in 2026. Previous treatment exposure and recurrent eyelid inflammation may have acted together to promote cumulative remodeling of the eyelid margin and meibomian glands. This multifactorial process may help explain the delayed recognition of distichiasis after years of periocular inflammatory activity. However, the retrospective photographs cannot establish the exact time of onset, and the absence of prospectively recorded lash counts, corneal staining, or meibomian gland imaging limits phenotypic characterization. Most importantly, a single observation cannot distinguish an effect of dermatomyositis-related inflammation from that of recurrent blepharitis, remote docetaxel exposure, or their combination. The proposed association should therefore be regarded as hypothesis-generating rather than causal.

The clinical manifestations of acquired distichiasis depend on the number, thickness, orientation, and corneal contact of the accessory eyelashes. Distichiatic lashes may remain asymptomatic or cause foreign-body sensation, irritation, photophobia, keratitis, and corneal ulceration [3,5]. In our patient, the accessory eyelashes were long and pigmented but were not associated with trichiasis, ocular symptoms, or corneal injury. Management should consequently be individualized based on symptom severity and ocular surface involvement. Available treatments include repeated epilation, electrolysis, cryotherapy, laser ablation, and surgical procedures for extensive or refractory disease [3,5,6]. In asymptomatic patients without corneal involvement, conservative management with regular ophthalmologic monitoring represents an appropriate approach.

## Conclusions

This case suggests a possible, hypothesis-generating association between chronic periocular inflammation and acquired distichiasis in a patient with dermatomyositis. It does not establish dermatomyositis as a cause, particularly because recurrent blepharitis and remote docetaxel exposure are plausible competing or contributing factors, and further observations are required to assess this relationship. Careful eyelid margin and ocular surface examination is important in patients with chronic periocular inflammation to identify potentially injurious lashes and guide treatment. In asymptomatic patients without corneal involvement, conservative management with regular ophthalmologic monitoring may be appropriate.
